# Estimated Prevalence of Risk Factors for Preeclampsia Among Individuals Giving Birth in the US in 2019

**DOI:** 10.1001/jamanetworkopen.2021.42343

**Published:** 2022-01-04

**Authors:** Sarahn M. Wheeler, Sabrena O. Myers, Geeta K. Swamy, Evan R. Myers

**Affiliations:** 1 Division of Maternal and Fetal Medicine, Department of Obstetrics and Gynecology, Duke University School of Medicine, Durham, North Carolina; 2 Duke University School of Medicine, Durham, North Carolina; 3 Division of Women’s Community and Population Health, Department of Obstetrics and Gynecology, Duke University School of Medicine, Durham, North Carolina

## Abstract

**Importance:**

Low-dose aspirin (LDA) is one of the few evidence-based interventions for preventing preeclampsia, which is a leading cause of maternal or fetal morbidity and mortality. Current guidelines recommend LDA based on the presence of risk factors for preeclampsia, but the population-based prevalence of these factors is unknown.

**Objective:**

To estimate population-level prevalence of preeclampsia risk factors used in prophylactic LDA guidelines for pregnant patients and the association of these risk factors with reported rates of pregnancy-related hypertension.

**Design, Setting, and Participants:**

A retrospective cohort study was conducted using National Center for Health Statistics birth certificate data to describe the frequency of pregnant individuals with moderate to high-risk factors for preeclampsia and pregnancy-related hypertension rates. The study used all birth records in the United States for the 2019 calendar year.

**Exposures:**

Documentation of preeclampsia risk factors: multifetal gestation, pregestational diabetes, chronic hypertension (high-risk factors) and nulliparity, a body mass index greater than 30, African American race, a maternal age 35 years or older, an interval of more than 10-years since last birth, and having low socioeconomic status (moderate risk factors).

**Main Outcomes and Measures:**

Prevalence of each risk factor alone and in combinations leading to a recommendation for LDA; incidence of pregnancy-related hypertension by risk factor and combinations of risk factors.

**Results:**

There were 3 695 019 pregnancies in 2019, including 528 778 with no risk factors, 169 540 with 1 or more high-risk factors, and 2 996 701 with 1 or more moderate-risk factors. The mean (SD) of the cohort was 29.1 (5.8) years. Multifetal gestation was the most common high-risk factor and found in 123 995 pregnancies (3.4%), and low socioeconomic status was the most common moderate-risk factor and present in 1 732 729 pregnancies (46.9%). Based on 2021 criteria (a single high- or moderate-risk factors for preeclampsia), 3 166 241 pregnant patients (85.7%) were eligible for LDA. The incidence of pregnancy-related hypertension increased with the number of moderate-risk factors. The 2021 guidelines suggest considering or recommend LDA in 92.3% of pregnancies diagnosed with pregnancy-related hypertension.

**Conclusions and Relevance:**

These data support the recently published guidelines and suggest further simplified guidelines recommending LDA to patients with any single moderate-risk factors.

## Key Points

**Question:**

What is the prevalence of preeclampsia risk factors used in prophylactic low-dose aspirin guidelines for pregnant patients?

**Findings:**

In this cohort study of all 3 695 019 recorded deliveries in the US in 2019; 4.5% of deliveries had 1 or more high-risk factors and 81.1% of deliveries had 1 or more moderate risks for preeclampsia. Pregnant patients meeting 2021 criteria to recommend or consider low-dose aspirin accounted for 85.7% of births and 92.3% of documented pregnancy-related hypertension in 2019.

**Meaning:**

Simplified guidelines recommending LDA to patients with any single moderate-risk factor or a universal approach warrant further consideration.

## Teaser

This cohort study estimates the population-level prevalence of preeclampsia risk factors used in prophylactic low-dose aspirin guidelines for pregnant patients and the association of these risk factors with reported rates of pregnancy-related hypertension.

## Introduction

Preeclampsia is a systemic hypertensive condition marked by elevated blood pressures and proteinuria that is unique to pregnancy. Preeclampsia increases the risk of fetal growth restriction, preterm birth, Cesarean delivery, long-term maternal cardiovascular disease, and is a leading cause of maternal and fetal morbidity and mortality.^1^ In a recent meta-analysis of 16 clinical trials, low-dose aspirin (LDA) reduced the risk of preeclampsia by 15%,^1,2,3^ especially when it was started before 16 weeks’ gestation. The United States Preventive Services Task Force (USPSTF) recommends LDA (81 mg/d) for the prevention of preeclampsia in individuals with a preeclampsia risk of 8% or higher, based on the presence of certain known risk factors.^4^

The 2014 USPSTF are complex, with a definite recommendation for LDA if 1 or more of 6 high-risk factors and consideration of LDA 2 or more of 11 moderate-risk factors are present. The recently updated 2021 guidelines are similarly complex. Similar to 2014, LDA is definitively recommended if 1 or more high-risk factors is present. The 2021 guidelines now recommend for LDA for a combination of moderate-risk factors and encourage consideration if a single moderate-risk factor is present. Given the continued complexity of these recent guidelines, the overall safety of LDA in pregnancy, and the potential consequences of preeclampsia for maternal and fetal outcomes, some researchers have argued for universal use of LDA in all pregnant individuals.^5,6^

Direct population-based data estimating the number of pregnant individuals who are candidates for LDA based on USPSTF guidelines and correlation with pregnancy-related hypertension outcomes are lacking. This study was conducted to determine population-level estimates of specific risk factors for preeclampsia and LDA candidacy.

## Methods

This retrospective cohort study uses publicly available birth certificate data from the National Center for Health Statistics (NCHS) calendar year 2019.^7^ The study protocol was approved by the institutional review board at Duke University and informed consent was waived because deidentified publicly available data were used in the analysis. This study followed the Strengthening the Reporting of Observational Studies in Epidemiology (STROBE) reporting guideline.

All recorded births from 2019 were included in the analysis. The exposures of interest were high and moderate-risk factors for preeclampsia based on the USPSTF guidelines.^1,4^ The standard birth certificate records 3 of the 6 high-risk factors for which LDA is definitively recommended: multifetal gestation, pregestational diabetes, and chronic hypertension. The remaining 3 high-risk factors—preeclampsia in a prior pregnancy, renal disease, and autoimmune disease—are not recorded in birth records. Among individuals without a documented high-risk factor, we assessed moderate-risk factors, including nulliparity, body mass index (BMI, calculated as weight in kilograms divided by height in meters squared) over 30, African American race, maternal age 35 years or older, more than a 10-year interval since last birth and low socioeconomic status (SES, defined by Medicaid as primary payer or participation in the Women, Infant, and Children [WIC] nutritional supplement program). The 2021 USPSTF guidelines also include in-vitro conception as a moderate-risk factor and the NCHS data captures assisted reproductive technology, which includes in vitro fertilization and intracytoplasmic sperm injection. The USPSTF guidelines include data on the African American race, thus that data was collected and presented in the results. Having a family history of preeclampsia, personal history of low birth weight, and being small for gestational age are moderate-risk factors that are not captured in birth records. The primary outcomes were the frequencies of each risk factor alone and in combinations that would lead to recommendations for LDA. For estimation purposes, we assumed that missing data on a given risk factor meant that the factor was not present.

LDA is most effective when started before 16 weeks’ gestation; therefore, we examined the frequency of patients meeting LDA criteria who initiated prenatal care by 16 weeks’ gestation. Although preeclampsia is not specifically recorded on the birth certificate, gestational hypertension (gHTN, including pregnancy-related blood pressure elevation and preeclampsia), and eclampsia (ie, seizures associated with preeclampsia) are recorded. We estimated the proportion of individuals with different combinations of risk factors who developed gHTN or eclampsia, collectively termed pregnancy-related hypertension. We estimated a number needed to treat (NNT) assuming LDA reduces overall pregnancy-related hypertension by an equivalent amount relative to the estimated relative risk reduction in preeclampsia used for developing the current guidelines of 0.85.^1^ No formal statistical testing was done, however, we calculated 95% CIs assuming a range of preeclampsia reduction from 0.75 to 0.95 consistent with the 2021 guidelines.^1^ All analyses were performed in JMP, version 15 (SAS Institute) and conducted from July to November 2021.

## Results

There were 3 695 019 pregnancies that resulted in a live birth in 2019. The mean (SD) age of the cohort was 29.1 (5.8) years. There were 528 778 (14.3%) that had no documented high or moderate-risk factors. One or more high-risk factors was recorded for 169 540 (4.5%) of births. Multifetal gestation (123 995 [3.4%]) was the most common high-risk factor.

Excluding the births with documented high-risk factors, 1 303 890 births (35.2%) with a single moderate-risk factor and 1 692 811 (45.8%) with 2 or more moderate-risk factors. The most common moderate-risk factors were low SES (1 732 729 [46.9%]), nulliparity (1 115 780 [30.2%]), and obesity (1 013 833 [27.4%]). Of individuals with 2 or more moderate-risk factors (1 664 088 of 3 695 019 births [45%]), low SES was 1 of the factors in 1 154 877 (69.4%) of cases. Based on 2021 guidelines when LDA is definitively recommended (ie, a single high-risk or a combination of 2 or more moderate-risk factors for preeclampsia), 1 862 351 (50.4%) of pregnant patients were eligible for LDA. Prenatal care was initiated at or before 16 weeks among 148 347 (87.5%) of patients with a single high-risk factor and 1 381 602 (83.0%) of patients with 1 or more moderate-risk factors.

The risk of gestational hypertension or eclampsia increased with the number of moderate-risk factors (Table 1). Birth records meeting 2021 USPSTF criteria to definitively recommend LDA accounted for 196 377 (65.3%) pregnancy-related hypertension, while records with only a single moderate-risk factor accounted for another 81 383 of 300 080 (27%) cases. The absolute number of gestational hypertension cases is similar among patients with no risk factors (23 188 of 300 080 [7.7%]) and patients with 1 or more high-risk factors (25 348 of 300 080 [8.4%]) (Figure). The recorded incidence of gestational hypertension in individuals with at least 2 moderate-risk factors (277 760 of 3 166 241 [8.8%]) is similar to the incidence used to estimate threshold NNT for the USPSTF guidelines.^4^ Assuming a relative risk of 0.85 used in the 2021 USPSTF guidelines, the NNT for 1 or more high-risk factors or 2 or more moderate-risk factors is 63 (95% CI, 38-190). Individuals with only 1 moderate-risk factor accounted for another 81 383 [27%]) of cases, with a pregnancy-related hypertension incidence of 6.3%. The NNT when LDA is recommended for any moderate- or high-risk factor is 76 (95% CI, 46-228). In the setting of universal LDA the NNT is 82 (95% CI, 49-246) (Table 2).

**Table 1.** Prevalence of Risk Factors and Gestational Hypertension and Eclampsia Outcomes

Risk Factors	Pregnancies with risk factors, No.	gHTN and eclampsia, No.	gHTN and eclampsia, %		NNT, relative risk (95% CI)^c^
			Rate^a^	Proportion^b^	
Total pregnancies	3 695 019	300 080	8.1	100.0	78 (47-233)
None	528 778	23 188	4.4	7.7	152 (81-456)
≥1 High-risk factors	169 540	25 348	15.0	8.4	45 (27-134)
Any 1 moderate-risk factors	1 303 890	81 383	6.3	27.0	107 (64-320)
Single moderate-risk factors					
Low socioeconomic status	465 804	18 441	4.0	6.1	168 (101-505)
Nulliparous	386 523	29 481	7.6	9.8	87 (52-262)
Age ≥ 35 y	204 718	9477	4.6	3.1	144 (86-432)
BMI ≥ 30	173 810	19 265	11.0	6.4	60 (36-180)
African American race	40 090	2207	5.5	0.7	121 (73-363)
Prior preterm birth	18 268	1492	8.2	0.5	82 (49-245)
≥ 10 y since last birth	9679	560	5.9	0.2	115 (69-346)
In vitro conception^d^	4998	457	9.1	0.2	73 (52-262)
Moderate-risk factors combined, No.					
2	1 109 585	98 292	8.9	32.7	75 (45-226)
3	474 696	55 747	11.7	18.5	57 (34-170)
4	99 372	15 297	15.4	5.1	43 (26-130)
5	8792	1615	18.4	0.5	36 (22-109)
6	367	78	21.3	0.02	31 (19-34)

Abbreviations: BMI, body mass index (calculated as weight in kilograms divided by height in meters squared); gHTN, gestational hypertension; NNT, number needed to treat; USPSTF, United States Preventive Services Task Force.

^a^ Among those with given risk factor or number of risk factors.

^b^ Among all cases of gestational hypertension or eclampsia.

^c^ Assumes 0.85 (95% CI, 0.75-0.95) relative risk for gestational hypertension, which is equivalent to USPSTF estimate for preeclampsia.

^d^ Newly added to the 2021 USPSTF Aspirin Use to Prevent Preeclampsia Guidelines.

**Figure.**
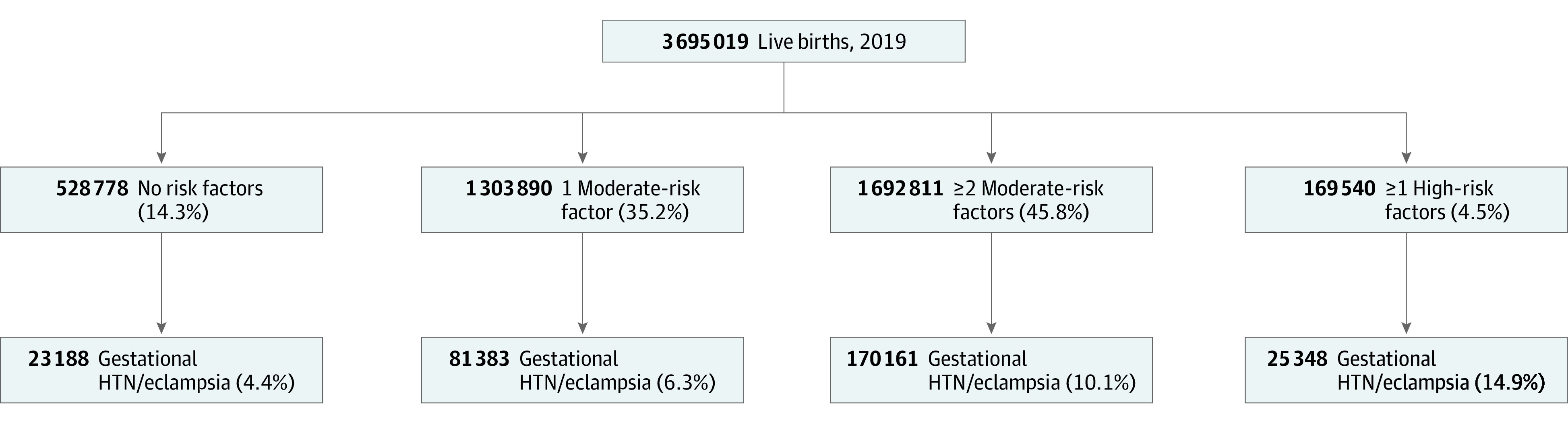
Preeclampsia Risk Factors and Gestational Hypertension (HTN) and Eclampsia Outcomes

**Table 2.** Comparison of Low-Dose Aspirin Strategies for Preeclampsia Prevention

LDA strategy	No.	PIH	%		Cases prevented (95% CI)	NNT (95% CI)
			PIH rate	All PIH		
**LDA given when recommended by 2021 USPSTF guidelines**						
LDA for ≥1 high-risk factor or ≥2 moderate-risk factors	1 862 351	196 377	10.5	65.3	29 457 (9189-49 094)	63 (38-190)
1 moderate- no risk factors (no LDA)	1 832 668	104 571	5.7	34.7		
**LDA given when considered by 2021 USPSTF guidelines**						
LDA for any moderate- high-risk factor	3 166 241	277 760	8.8	92.3	41 664 (13 888-69 440)	76 (46-228)
No risk factors (no LDA)	528 778	23 188	4.4	7.7		
**Universal LDA**						
LDA for all	3 695 019	300 948	8.1	100	45 142 (15 047-75 237)	82 (49-246)

Abbreviations: LDA, low-dose aspirin; NNT, number needed to treat (assumes RR 0.85 [95% CI, 0.75-0.95]); PIH, pregnancy-related hypertension (ie, gestational hypertension and preeclampsia); USPSTF, United States Preventive Services Task Force.

## Discussion

In 2019, more than 85% of pregnant individuals met the 2021 USPSTF criteria to recommend or consider LDA to prevent preeclampsia. More than 80% who met these criteria presented for prenatal care within the optimal time-period to initiate therapy. Birth records with documented conditions meeting current guidelines for LDA account for almost two-thirds of cases of gestational hypertension and eclampsia.

The single most commonly documented risk factor was low SES. Studies documenting an increased risk of preeclampsia with low SES use varying definitions. We used Medicaid as primary payer and participation in the WIC nutritional supplement program as markers of low SES, however the eligibility criterion for these programs varies by state. These strict criteria likely underestimate the extent of patients with low SES. Even with the likely underestimation, we observed a 7.8% and 4% prevalence of pregnancy-related hypertension with low SES overall and low SES in isolation, suggesting the clinical importance of this factor.

The African American race is also considered a moderate-risk factor, and there is a growing understanding that race is not a genetic or biological construct, but rather race measures an individual’s lived experiences with racism and discrimination.^8^ Although self-report is the criterion standard^9^ and there is likely some variation in how race is captured across hospitals, studies suggest that birth record data are highly accurate in race reporting compared with direct patient interviews.^10^ The inclusion of SES and race in the USPSTF guidelines highlights the importance of ensuring clinicians ascertain data about these and other social determinants of health with a clear understanding that health inequities rather than biological differences drive these associations.

In vitro conception was added to the list of moderate-risk factors for preeclampsia in the 2021 guidelines. The birth record documents in vitro conception, however, the literature suggests under-reporting is common.^11,12^ We strongly suspect under-reporting in the 2019 birth record data because only 49 000 births with in-vitro conception are recorded in the birth certificate data accounting for 66% of the births in the Society for Assisted Reproductive Technology database. Although the birth record data likely underestimate the cases of in-vitro conception, there is likely a limited impact on our estimates of individuals meeting the criteria for LDA as these individuals are more likely to be advanced maternal age or have other comorbidities well documented in the birth record data.

Our analysis questions the necessity for the relative complexity in the 2021 LDA guidelines. Wed demonstrated that the NNT for individuals who meet the 2021 criteria to recommend LDA (≥2 moderate-risk factors or ≥1 high-risk factor) is 63. For a single moderate-risk factor when LDA is considered, the NNT increases modestly to 76. In the setting of universal treatment, the NNT is 82. These data suggest the incremental difference in NNT from consideration of LDA to universal LDA is 6. Several model-based analyses suggest that both the clinical harm-benefit ratio and cost-effectiveness of universal LDA are favorable compared to risk-factor based strategies given the risks associated with preeclampsia and the low cost and favorable safety profile of LDA.^5,13,14^ Similarly complex risk stratification protocols for influenza vaccination were abandoned because of their complexity and lead to universal vaccination guidelines.^15^

### Limitations

Although our findings add to the growing conversation about strategies to maximize LDA in pregnancy, several important limitations must be considered. Birth record data are entered by hand and subject to errors in reporting. Literature suggests birth record data accurately capture pregnancy-related hypertension yet may underestimate chronic hypertension.^16^ Birth records also do not record several high and moderate-risk factors, which may have undercounted how many patients meet the criteria for LDA. The omission of these risk factors suggests we have underestimated the proportions of patients that meet LDA criteria. However, many of the conditions not captured are relatively rare (autoimmune and renal disease) or are rarely captured in the medical record (family history of preeclampsia), suggesting a limited impact on the estimates presented. Approximately 20% of patients with a personal history of preeclampsia have recurrent preeclampsia in a future pregnancy. It is unclear whether a personal history of preeclampsia influences the incidence of future pregnancy; therefore, estimating the impact of this omission is challenging. Furthermore, the prevalence of the high- and moderate-risk factors are not fixed, and many risk factors are increasing in prevalence. Based on likely increases in many of the risk factors over time, we suspect the current NNT estimates are likely overestimations.

The birth record documents pregnancy-related hypertension and is inclusive of individuals with gHTN who do not meet strict preeclampsia criteria. However, this broader definition is unlikely to have a meaningful impact on our estimates because gHTN alone is associated with adverse outcomes, and there is progression to preeclampsia in up to 50% of cases. ACOG suggests the distinction between gHTN and preeclampsia is “an exercise of nomenclature rather than a pragmatic one.”^17^

Reliance on birth certificate data limits our ability to incorporate clinician recommendations and patient adherence to LDA guidelines. It is possible that the cases of gHTN and eclampsia observed in the population level data were in the setting of LDA therapy. However, the incidence of pregnancy-related hypertension was similar among individuals with late prenatal care (7.4%) compared with those starting prenatal care prior to 16 weeks (8.1%). There are data suggesting patient nonadherence to LDA is associated with pill burden and poor communication with providers.^18^ As dosing strategies are reevaluated, further investigations including patient perceptions, provider adherence to guidelines, and patient-provider communication will be critical.

## Conclusions

Preeclampsia is an important cause of maternal and fetal morbidity and mortality, and LDA is one of very few evidence-based preventions. Given the high prevalence of risk factors for preeclampsia and the complexity of existing guidelines, further consideration of expanding definitive LDA recommendations to individuals with at least 1 moderate-risk factor, or simply universal LDA for all pregnant individuals, is needed.
